# Mechanisms that link circadian preference to problematic smartphone and social media use in young adults

**DOI:** 10.1371/journal.pone.0331961

**Published:** 2025-09-12

**Authors:** Anna-Stiina Wallinheimo, Simon L. Evans

**Affiliations:** 1 School of Psychology, Sport, and Health Sciences, University of Portsmouth, Portsmouth, United Kingdom; 2 School of Psychology, University of Surrey, Guildford, United Kingdom; Arba Minch University, ETHIOPIA

## Abstract

Evening-types are at higher risk of problematic smartphone use and addiction to social media, but little is known about the possible mediating factors. Given the rising prevalence and broad negative impacts of smartphone and social media addiction, these factors require identification. Young adults (*N* = 407) aged 18–25, with an average age of 19.8 years, completed a battery of validated measures online. We tested mental health (anxiety and depression symptoms), loneliness, and poorer sleep quality as potential mediators in the relationships between eveningness and problematic smartphone use and social media addiction. As expected, evening types had higher prevalence of problematic smartphone use and social media addiction. Eveningness was also associated with higher anxiety and depression symptoms, loneliness, and poorer sleep quality. Two separate parallel mediation analyses were then conducted, with these three factors entered simultaneously as mediators. For problematic smartphone use, a partial mediation occurred, with loneliness as the significant mediating variable. For social media addiction, both loneliness and anxiety were significant mediators, and a full mediation was found. These important findings point to loneliness and anxiety as crucial explanatory variables for problematic technology use in young adults, suggesting that young adult evening types resort to smartphone/social media use as a dysfunctional coping strategy for loneliness and anxiety. Given the prevalence of problematic smartphone use and social media addiction amongst young people worldwide, and their wide-ranging negative impacts, this has important implications for prevention and intervention strategies to enhance young adults’ mental health, functioning, and well-being.

## Introduction

Although smartphones and social networking services have brought about numerous benefits, there is a growing concern about their addictive potential [1]. Problematic smartphone use (PSU) refers to a dysfunctional relationship with smartphones, for example, feelings of anxiety when the smartphone is not accessible or neglecting other activities because of smartphone use [2], negatively impacting daily functioning and life satisfaction [3,4]. PSU is prevalent among adolescents and young adults [5]. Likewise, social media addiction, where users devote so much time and effort to social media that they neglect other responsibilities and cannot regulate their usage [6] affects nearly 40% of the UK student-age population [7], with female students at higher risk [8]. Given the prevalence and deleterious consequences of PSU and social media addiction in young adults, it is important to determine the risk factors and mechanisms involved.

Circadian preference is one of these risk factors. Circadian or diurnal preference denotes a person’s favoured times for daily activities, including waking up and going to bed. This preference spans a spectrum between two extremes: morning types, who like to rise and sleep early, and evening types, who favour later hours for both [9,10]. Eveningness prevalence is highest in late adolescence [11], coinciding with the transition into university life: up to 40% of university students identify as evening types [12,13]. Evening types show higher rates of addictive behaviours [14], including PSU and social media addiction, as demonstrated across samples from a variety of countries [15–19]. For example, amongst university students, evening circadian preference has been shown to be a very strong predictor of PSU, over and above personality traits as measured by the Big Five Inventory [17], while Blachnio et al., (2015) identified more intensive and intrusive use of social media platforms among young adult evening types. However, we can identify no studies investigating mediating factors in this relationship; thus, the current study addresses this knowledge gap.

As well as higher risk of addictive behaviours, eveningness has also been reliably linked to poorer sleep quality [20] and mental health symptomology [21–24]. Evening types are more susceptible to depression and anxiety symptoms, and this has been comprehensively demonstrated in young adults [25,26]. ‘Social jetlag’ in evening types (the discrepancy between the timings dictated by their internal body clock and those of external social obligations) also contributes to their higher risk of adverse mental outcomes and impaired sleep [27]. Smartphone use immediately before and after going to bed is common among young people and is well known to affect sleep negatively [28–30]. However, sleep problems are also predictors of problematic technology use, as shown by a longitudinal study in which difficulties initiating and staying asleep were predictive factors of internet addiction [31]. Thus, the sleep difficulties experienced by many evening types may well, in turn, lead to a greater dependence on smartphones and social media as a dysfunctional coping mechanism for their insomnia, leading to further harmful effects on their sleep timings and quality. Sleep quality is therefore a strong candidate mechanism that could mediate the relationship between eveningness and PSU/social media addiction.

Other strong candidate mechanisms are loneliness and mental health symptomology. Eveningness has been linked to a higher risk of loneliness in large studies of both young [26] and mid-age (40–70 years) adults [21]. This might be explained by a lack of social companionship outside the regular daily routines, leading to heightened feelings of isolation and loneliness at night. Eveningness has also been reliably linked to the risk of poorer mental health [21–23]; evening types are more susceptible to depression and anxiety symptoms, and this has been comprehensively demonstrated in young adults [25,26]. Loneliness and poor mental health could mediate the association between eveningness and PSU/social media addiction since numerous studies suggest that these constitute risk factors for PSU and social media addiction [32]. Review articles and a recent meta-analysis report that PSU is robustly associated with both anxiety and depression, with the authors arguing that, in terms of causality, depressive and anxious symptoms make people more vulnerable to PSU as a form of maladaptive coping mechanism [5,33]. Consistent with this, a longitudinal study in young adults showed that, over time, trajectories of escalating depression and anxiety go hand in hand with a greater risk of developing internet addiction [34]. Regarding loneliness, more lonely individuals tend to rely on smartphones and social media to try, and fulfil their relationship needs [35], and as a strategy to reduce boredom and loneliness [36]. When feelings of anxiety, depression, and/or loneliness are experienced, smartphones and social media are used as a distraction, and as a form of escape from negative emotions [37]. However, this can bring about a negative feedback loop; the resulting social monitoring can exacerbate feelings of depression and anxiety as users compare themselves negatively to others [38,39]. In addition, studies suggest that mental health problems (depressive symptoms in particular) can make individuals more impulsive by impairing prefrontal cortex executive control functions, raising the risk of addictive behaviour [40,41] and thus providing another mechanism linking poor mental health to enhanced risk of PSU and social media addiction.

In sum, the association between eveningness and the risk of addiction to smartphones and social media has been well demonstrated in the literature [15–17]. Eveningness has also reliably been linked to poorer sleep quality, higher mental health symptomology, and loneliness, which are factors that raise the risk of PSU and social media addiction and so could potentially act as mediators: this is the focus of the current study. Additionally, we acknowledge that social media platforms may deliberately exploit attention and reward systems to engage users [42], and that blue light exposure, especially before bedtime, can negatively affect circadian rhythms and sleep quality [43]. However, further investigations into these areas are outside the scope of our research. We hypothesised that eveningness would be associated with higher anxiety and depression symptoms, loneliness, and poorer sleep quality, as well as with increased PSU and social media addiction. We then explored (using mediation models amongst cross-sectional survey data from young adults) which of these factors might mediate the relationships between (1) eveningness and PSU and (2) eveningness and social media addiction.

## Methods

### Participants

The current study’s inclusion criteria were undergraduates aged 18–30 years. After excluding missing/incomplete responses, this resulted in a sample size of *N* = 407 (*M*age = 19.80 years, *SD* = 1.54 years). The participants’ ages ranged from 18 to 25 years. There were 238 males and 159 females. Ten participants did not disclose their gender.

### Materials

#### Smartphone application-based addiction scale (SABAS).

SABAS measures levels of PSU [44]. SABAS comprises six questions about smartphone use habits (e.g., ‘My smartphone is the most important thing in my life’; ‘If I cannot use or access my smartphone when I feel like, I feel sad, moody, or irritable’), based on a Likert scale from 1 (strongly disagree) to 6 (strongly agree). The total score is calculated by summing these, leading to a minimum score of 6 and a maximum score of 36.

#### Bergen social media addiction scale (BSMAS).

BSMAS measures social media addiction levels [45]. The scale contains six items covering core addiction elements [46]: salience (importance in a person’s life), mood modification (feelings of escape or numbing that the addiction provides), tolerance (an individual’s need to increase the size of their ‘fix’), withdrawal symptoms, conflict (between the addict and those around them), and relapse (the tendency for repeated reversions to earlier patterns). The questions are rated on a scale of 1–5, with 1 meaning ‘very rare’ and 5 meaning ‘very often’. The questions (e.g., ‘You spend a lot of time thinking about social media or planning how to use it.’; ‘You feel an urge to use social media more and more.’) measure the level of preoccupation with social media, including the amount of time spent thinking about or planning to use social media, the urge to use it more, using social media to forget personal problems, unsuccessful attempts to reduce usage, feeling uneasy when prohibited from using it, and the impact of excessive use on work or studies. The total score on the BSMAS ranges from 6 to 30, with higher scores indicating a greater problematic social media use.

#### Hospital anxiety and depression scale (HADS).

HADS measures anxiety (HADS-A) and depression (HADS-D) within the last week [47]. It is a 14-item scale, with seven items relating to anxiety (‘I feel tense or wound up’; ‘I get a sort of frightened feeling as if something awful is about to happen’; ‘Worrying thoughts go through my mind’) and seven relating to depression (e.g., ‘I still enjoy the things I used to enjoy’; ‘I can laugh and see the funny side of things’; I have lost interest in my appearance’) on a 4-point scale (0–3) with subscale totals ranging from 0–21.

#### De Jong Gierveld Loneliness Scale.

De Jong Gierveld Loneliness Scale is a 6-item scale for overall, emotional, and social loneliness. Three questions (e.g., ‘I experience a general sense of emptiness’; I miss having people around me’) measure emotional loneliness and another three (e.g., ‘There are plenty of people I can rely on when I have problems’; ‘There are many people I can trust completely’) measure social loneliness. There are three response categories to answer these questions: ‘Yes’, ‘More or less’, and ‘No’, giving a sum in the range of 0 (least lonely) to 6 (most lonely) [48].

#### Pittsburgh sleep quality index (PSQI).

PSQI measures subjective sleep quality [49]. It comprises seven components that assess various aspects of sleep, including subjective sleep quality, sleep latency, sleep duration, sleep efficiency, sleep disturbances, use of medication, and daytime dysfunction. The total PSQI score ranges from 0 to 21, with higher scores indicating poorer sleep quality. The PSQI is highly accurate in detecting insomnia, with a threshold of > 5 recommended for young adults [50].

### Horne and Östberg Morningness-Eveningness Questionnaire: A reduced scale (rMEQ)

rMEQ is the most commonly used method to assess circadian preference, with five questions on a Likert scale [1–6], e.g., ‘Considering only your own diurnal rhythm, at what time would you get up if you were entirely free to plan your day?’; ‘At what time in the evening do you feel tired and, as a result in need of sleep?’ [51]. Total scores for the rMEQ range from 4 to 26; a higher score indicates greater morningness preference, categorised as eveningness: < 12; neither: 12–17; morning: > 17.

### Procedure

The University of Surrey Ethics Committee approved the present study. It was advertised to all undergraduate students at Surrey (no exclusion criteria) via the online research participation portal. The data collection period lasted from April 16, 2021, to March 30, 2023. All participants provided written consent before completing an online Qualtrics survey that initially gathered demographic data, followed by the SABAS, BSMAS, HADS, loneliness, PSQI, and rMEQ measures. Participants were then debriefed and thanked for their participation. They received course credits for their involvement.

### Analytic plan

First, we split the sample by circadian preference category based on their rMEQ scores, and performed ANOVAS to test for group effects on SABAS, BSMAS, HADS-A, HADS-D, loneliness, and PSQI. We then tested for interrelationships between circadian preference as a continuous variable (raw rMEQ score) and the variables of interest. Two separate parallel mediation analyses then followed, using Hayes’ (2013) Process Macro (model 4), with 5000 bootstrap samples, to examine whether the effect of circadian preference (as a continuous variable) on (1) SABAS and (2) BSMAS, was mediated by loneliness, anxiety, depression, and/or poor sleep quality. Thus, the outcome variable for the mediation analysis was SABAS or BSMAS, with raw rMEQ score as the predictor variable. Loneliness (M1), HADS-A (M2), HADS-D (M3), and PSQI (M4) were the mediators. We did not consider gender as a covariate, because previous studies have shown similar patterns across genders in PSU [18].

## Results

Before conducting any statistical analyses, we conducted normality checks: Shapiro – Wilk test indicated that all the variables were normally distributed. The Shapiro-Wilk test was used due to its power in checking the assumption of normality in a data set, particularly with small to moderate sample sizes. We also viewed histograms and checked box plots, but found no extreme values or outliers. For descriptive statistics, see Table 1. The participants were classified as either morning, intermediate, or evening circadian preferences based on their rMEQ score. The morning circadian preference group (7.4%) comprised 30 participants (*M*age = 20.13 years, *SD* = 1.53 years). In the intermediate circadian preference group (46.7%), there were 190 participants (*M*age = 19.92 years, *SD* = 1.54 years), while the evening circadian preference group (45.9%) comprised 187 participants (*M*age = 19.62 years, *SD* = 1.54 years).

**Table 1:** Descriptive statistics for the outcome variables.

	*N*	*M*	*SE*
**SABAS**	407	19.07	.28
**BSMAS**	407	15.62	.25
**HADS-A**	407	10.39	.20
**HADS-D**	407	6.4	.18
**Loneliness**	407	3.53	.09
**rMEQ**	407	11.87	.19
**PSQI**	407	7.43	.17

SABAS = Smartphone Application-Based Addiction Scale; BSMAS = Bergen Social Media Addiction Scale; HADS-A = Hospital **Anxiety** and Depression Scale; HADS-D = Hospital Anxiety and **Depression** Scale; Loneliness = De Jong Gierveld Loneliness Scale; rMEQ = Reduced Morningness-Eveningness Questionnaire; PSQI = Pittsburgh Sleep Quality Index.

### ANOVAs

ANOVAs were conducted to test for the effects of the circadian preference category on the variables of interest (see Table 2). On SABAS, there was a significant effect of circadian preference category, *F*(2, 404) = 7.63, *p* < 001, η^2^ = .04. Pairwise post-hoc comparisons indicated differences between the morning and evening groups, *p* < .001, and between intermediate and evening groups, *p* = .04. On BSMAS there was no significant effect of circadian preference group *F*(2, 404) = 1.98, *p* = .14, η^2^ = .01.

**Table 2:** Means and standard errors for the outcome variables, by circadian preference category.

	Morning *n* = 30		Intermediate *n* = 190		Evening *n* = 187	
	*M*	*SE*	*M*	*SE*	*M*	*SE*
**SABAS**	16.13*	1.0	18.61*	.40	20.01*	.40
**BSMAS**	14.53	.93	15.3	.37	16.12	.37
**HADS-A**	9.97	.74	9.98	.30	10.87	.30
**HADS-D**	6.33	.65	5.83*	.26	6.99*	.26
**Loneliness**	3.13	.34	3.38	.14	3.73	.14
**PSQI**	6.17*	.61	6.93*	.24	8.15*	.24

SABAS = Smartphone Application-Based Addiction Scale; BSMAS = Bergen Social Media Addiction Scale; HADS-A = Hospital **Anxiety** and Depression Scale; HADS-D = Hospital Anxiety and **Depression** Scale; Loneliness = De Jong Gierveld Loneliness Scale; rMEQ = Reduced Morningness-Eveningness Questionnaire; PSQI = Pittsburgh Sleep Quality Index. * = indicates significance at the 0.05 level between the different circadian preference categories.

There was no circadian preference effect on HADS-A, *F*(2, 404) = 2.45, *p* = .09, η^2^ = .01. On HADS-D, there was a statistically significant effect of circadian preference group, *F*(2, 404) = 5.07, *p* = .007, η^2^ = .02. Pairwise post-hoc comparisons indicated a significant difference between the intermediate and evening circadian preference groups only, *p* = .005. On the PSQI, there was a significant effect of circadian preference group *F*(2, 404) = 8.74, *p* < .001, η^2^ = .04, pairwise comparisons revealed a significant difference between morning and evening groups as well as between intermediate and evening groups at *p* < .001. However, for loneliness *F*(2, 404) = 2.37, *p* = .10, η^2^ = .01, the effect of the circadian preference group was not significant.

### Correlations

Prior to the mediation analyses, we explored intercorrelations between variables, with circadian preference as a continuous variable (raw rMEQ score). Pearson’s *r* correlations showed that all the variables were strongly correlated with each other (Table 3). Notably, there were significant correlations between rMEQ and HADS-A, SABAS, and loneliness, despite ANOVAS not finding effects of circadian preference category on these variables. Thus, we proceeded with the mediations.

**Table 3:** Correlations between the variables.

	SABAS	BSMAS	HADS-A	HADS-D	Loneliness	rMEQ	PSQI
**SABAS**	1						
**BSMAS**	.694**	1					
**HADS-A**	.178**	.185**	1				
**HADS-D**	.160**	.117*	.520**	1			
**Loneliness**	.218**	.195**	.333**	.324**	1		
**rMEQ**	−.210**	−.134**	−.144**	−.195**	−.162**	1	
**PSQI**	.157**	.129**	.462**	.429**	.294**	−.293**	1

SABAS = Smartphone Application-Based Addiction Scale; BSMAS = Bergen Social Media Addiction Scale; HADS-A = Hospital **Anxiety** and Depression Scale; HADS-D = Hospital Anxiety and **Depression** Scale; Loneliness = De Jong Gierveld Loneliness Scale; rMEQ = Reduced Morningness-Eveningness Questionnaire; PSQI = Pittsburgh Sleep Quality Index. ** correlation is significant at the 0.01 level; * correlation is significant at the 0.05 level.

### Mediation analyses

**Fig 1:**
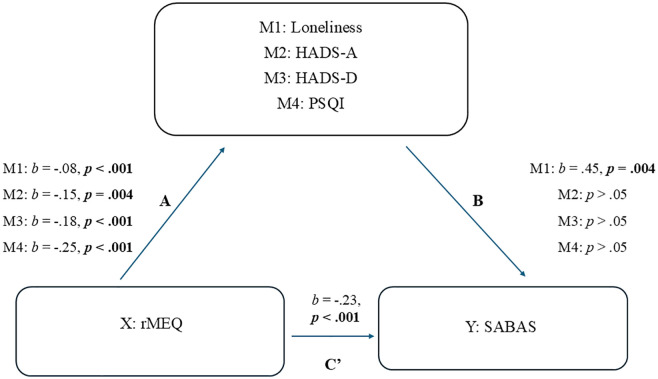
Mediation analysis with SABAS as the outcome variable.

Two separate parallel mediation analyses were conducted. For the first, the outcome variable was **SABAS** (smartphone addiction score), Loneliness (M1), HADS-A (M2), HADS-D (M3), and PSQI (M4) were the mediators, and rMEQ (as a continuous variable) was the predictor variable (Fig 1). All the paths (A) from rMEQ to the four mediators were significant. However, only **loneliness** was significant when looking at the paths (B) from the four mediators to SABAS. The mediating (indirect) effect was significant, clearly indicating that loneliness mediated the effects of the evening circadian preference on SABAS. Furthermore, the direct effect C’ was also significant. Thus, the path through loneliness **partly** mediated the effect of rMEQ on SABAS (Table 4).

**Table 4:** The mediating effect of loneliness in the relationship between rMEQ and SABAS.

Path (Loneliness)	Coefficient (*b*)	*p*	95% CI
X – M: A	−.08	<.001	[-.12 (LL) to -.03 (UL)]
M – Y: B	.45	.004	[.14 (LL) to.76 (UL)]
X – Y (Direct, controlling for M): C’	−.23	<.001	[-.38 (LL) to -.09 (UL)]
X – M – Y (Mediating, Indirect Effect)	−.03	<.05	[-.07 (LL) to -.009 (UL)]

**Fig 2:**
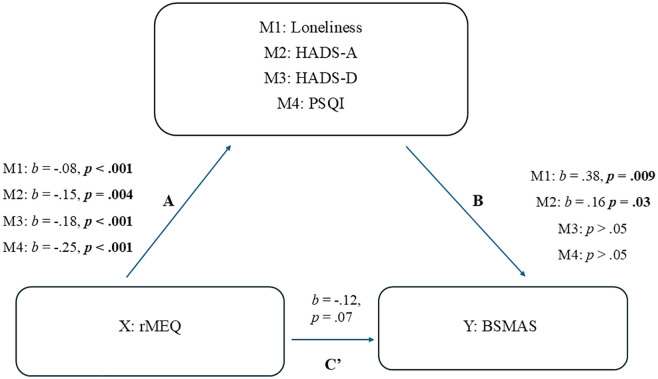
Mediation analysis with BSMAS as the outcome variable.

The second mediation analysis was the same as the first, except the outcome variable was **BSMAS** (social media addiction), (Fig 2). All the paths (A) from rMEQ to the four mediators were significant. However, only **loneliness** and **HADS-A** (path B) from the mediators to BSMAS were significant. When investigating the mediating (indirect) effect of the four mediators on BSMAS, only loneliness and HADS-A showed significance. However, C’ (direct effect) did not show significance. Thus, a **full** mediation occurred, as eveningness (rMEQ) did not have a direct significant effect on BSMAS with the mediators included (Tables 5 and 6).

**Table 5:** The mediating effect of loneliness in the relationship between rMEQ and BSMAS.

Path (Loneliness)	Coefficient (*b*)	*p*	95% CI
X – M: A	−.08	<.001	[-.12 (LL) to -.03 (UL)]
M – Y: B	.38	.009	[.10 (LL) to.66 (UL)]
X – Y (Direct, controlling for M): C’	−.12	.07	[-.25 (LL) to.008 (UL)]
X – M – Y (Mediating, Indirect Effect)	−.03	<.05	[-.06 (LL) to -.006 (UL)]

**Table 6:** The mediating effect of anxiety in the relationship between rMEQ and BSMAS.

Path (Anxiety)	Coefficient (*b*)	*p*	95% CI
X – M: A	−.15	.004	[-.25 (LL) to -.05 (UL)]
M – Y: B	.16	.03	[.01 (LL) to.31 (UL)]
X – Y (Direct, controlling for M): C’	−.12	.07	[-.25 (LL) to.008 (UL)]
X – M – Y (Mediating, Indirect Effect)	−.02	<.05	[-.06 (LL) to -.0003 (UL)]

## Discussion

In the present study, we explored, for the first time, the mechanisms which might explain the association between eveningness and the risk of addiction to smartphones and social media among young adults. Specifically, we considered mental health symptoms of anxiety and depression, loneliness, and poor sleep quality as mediators, as these have been consistently linked to both eveningness and risk of smartphone and social media addiction. Loneliness was found to partially mediate the effect of eveningness on smartphone addiction. In relation to social media addiction, both loneliness and anxiety were found to be significant mediators, and a full mediation occurred.

As expected, a main effect of the circadian preference group was found on sleep quality, depression, and PSU. Although we did not observe any circadian preference group effect on social media addiction, anxiety, or loneliness in the correlation analyses (with rMEQ as a continuous variable), we did find the expected relationships with these variables. The lack of group effects likely stemmed from mismatched circadian preference group sizes in the ANOVAs, as there were only 30 morning types in our sample, which meant a main effect could not be detected, but a low prevalence of morning types is typical of the age point under study.

Regarding PSU, we found that there was a **partial** mediation effect of **loneliness** on the relationship between eveningness and smartphone addiction. Loneliness was the only significant predictor, as there were no mediating effects of anxiety/depression symptoms or sleep quality. The link between eveningness and loneliness is in line with previous studies [21,52], and possibly due to a lack of companionship after regular university/social activities that might exacerbate feelings of loneliness at night time. They might then turn to smartphone use to fulfil their relationship needs [35], temporarily alleviating feelings of loneliness and helplessness [52]. It is also possible that smartphones were used simply as a distraction from loneliness, as a way to escape from the associated negative emotions [37]. While some previous studies [31] suggest a link between sleep problems and the subsequent development of internet addiction in childhood/adolescence, we did not find evidence of lower sleep quality acting as a mediator of eveningness on PSU cross-sectionally amongst our undergraduate-aged participants; rather, it was explicitly loneliness that had a mediating effect.

Further, when investigating loneliness, mental health symptoms, and poor sleep quality as potential mediators of the effects of eveningness on social media addiction, we found a **full** mediation occurred. L**oneliness** and **anxiety** were the significant mediators; loneliness was the stronger of the two. As with PSU it seems that evening-type students might be more vulnerable to social media addiction [5,33] as a response to their feelings of loneliness, but with anxiety also a factor when it comes to social media addiction. As with PSU, we found that it was these factors, rather than poor sleep, which explained why evening types were more likely to turn to social media: to connect with others due to a lack positive social interaction and emotional support, and/or as a way to manage anxiety – and in the case of social media addiction, these two factors fully mediated the relationship. However, as discussed previously, social media is often not a source of positive social support and may in fact increase anxiety due to negative social comparisons [38,39]. Thus, as a coping mechanism, it is likely ineffective and might exacerbate the feelings of anxiety and loneliness which the individual is trying to address.

Although outside the scope of the current study, there are various neurobiological and cognitive mechanisms which could be explored further in the context of future work. Eveningness is associated with alterations in dopaminergic activity, which is central in reward processing and addiction [14]. Additionally, delayed melatonin secretion and altered cortisol rhythms can affect mood regulation and the stress response, contributing to maladaptive behaviours including excessive digital technology use [53]. Also, evening-types are typically higher in impulsivity and sensation seeking, making them more susceptible to the instant gratification provided by smartphones and social media [11], especially during the late-night hours when cognitive control is lower [4]. Late-night smartphone use can also exacerbate sleep disruption through blue light exposure and cognitive stimulation, perpetuating a cycle of sleep loss and compensatory media use [43], although it should be noted that sleep quality was not found to act as a mediator in any of our analyses.

The current study has some clear limitations. The population comprised students only, which limits the generalisability of these findings. Additionally, self-reported questionnaires were used, so responses may have been affected by recall or social desirability biases. Additionally, any possible confounding factors (e.g., cultural or environmental influences) were outside the scope of this study. A larger study, perhaps using qualitative methods, could be used to investigate their potential effects. Due to the cross-sectional nature of this research, we cannot draw conclusions about the directionality of our findings. This type of design does have limitations in the current methodological literature. The use of longitudinal data to explore the relationships could have facilitated the establishment of a cause-and-effect relationship between our variables. However, this study aimed not to determine causality, but rather to understand the psychological mechanisms underlying this relationship.Future studies should employ a longitudinal design in order to make stronger inferences regarding causality, as well as exploring the role of the neurobiological and cognitive factors discussed above.

## Conclusions

The current study provides valuable insights, identifying loneliness as a key mediating factor in the effect of eveningness on both PSU and social media addiction, with anxiety also implicated. The fact that loneliness and anxiety fully mediated the relationship between eveningness and social media addiction is significant. This work addresses a significant knowledge gap, as this is the first study to explore what mediates the association between circadian preference and PSU/social media addiction.

As such, the findings provide vital new knowledge. The current findings imply that young adult evening types, amongst whom rates of PSU/social media addiction are very high, are likely resorting to these technology use habits as a means of addressing their anxiety and feelings of loneliness. Unfortunately, these habits are dysfunctional in that they often, in fact, exacerbate anxiety and a sense of isolation. Therefore, the implications of our findings for the well-being of young adults are significant. Young adults should be discouraged from turning to social media and smartphone use as coping mechanisms, and instead, be informed regarding effective strategies and interventions for addressing their loneliness and anxiety. Given the many adverse effects of PSU/social media addiction, alongside the mental health crisis engulfing young people worldwide [54]. The current study offers timely insights into what should be prioritised for effective intervention strategies, with tailored advice directed to young adult evening types, who are at highest risk.

## Supporting information

S1 File Revised raw data. (XLSX)

S2 File Metadata. (DOCX)
