# Supplementary material for: Mechanisms that link circadian preference to problematic smartphone and social media use in young adults
Source: PLoS One. 2025 Sep 12;20(9):e0331961. doi: 10.1371/journal.pone.0331961 (PMC12431251; doi:10.1371/journal.pone.0331961)
Supplement: S2 File — (DOCX) [file pone.0331961.s002.docx]

**Metadata:**

**SBJ:** This is the participant ID.

**Age:** This is the participant’s age in years.

**Categorical data:**

**Gender:** The male gender was coded as 1, and the female gender was coded as 2. Participants who did not confirm their gender were coded as 3.

**rMEQ_category:** The rMEQ category represents chronotype groups (evening, intermediate, or morning) based on the cut-off scores on rMEQ (total). Total scores for the rMEQ range from 4 to 26; a higher score indicates greater morningness preference, categorised as eveningness : < 12; neither: 12 – 17; morning: > 17. Evening types are coded as 1, intermediate as 2, and morning 3 in the raw data file.

**Continuous data:**

**1 HADS_Anxiety:** These are the **anxiety** scores based on the Hospital Anxiety and Depression Scale (HADS). The total scores range from 0 to 21.

**2 HADS_Depression:** The **depression** scores based on the Hospital Anxiety and Depression Scale (HADS). The total scores range from 0 to 21.

**3 Smartphone Application-Based Addiction Scale (SABAS):** SABAS measures problematic smartphone use. It comprises six questions about smartphone use habits. The total score is calculated by summing these, leading to a minimum score of 6 and a maximum score of 36.

**4 Bergen Social Media Addiction Scale (BSMAS):** The BSMAS measures social media addiction levels. It contains six items covering core addiction elements. The total score on the BSMAS ranges from 6 to 30, with higher scores indicating more problematic social media use.

**5 rMEQ_total:** These are the Horne and Östberg Morningness-Eveningness Questionnaire scores: A reduced scale (rMEQ). Total scores for the rMEQ range from 4 to 26; a higher score indicates greater morningness preference.

**6 Pittsburgh Sleep Quality Index (PSQI):** This index represents total sleep quality scores based on the Pittsburgh Sleep Quality Index (PSQI). The total PSQI score ranges from 0 to 21, with higher scores indicating poorer sleep quality.

**7 De Jong Gierveld Loneliness Scale:** De Jong Gierveld Loneliness Scale is a 6-item scale for overall, emotional, and social loneliness. There are three response categories to answer these questions: 'Yes', 'More or less', and 'No', giving a sum in the range of 0 (least lonely) to 6 (most lonely).
